# Botulinum Toxin A for Sialorrhoea Associated with Neurological Disorders: Evaluation of the Relationship between Effect of Treatment and the Number of Glands Treated

**DOI:** 10.3390/toxins10020055

**Published:** 2018-01-27

**Authors:** Domenico A. Restivo, Mariangela Panebianco, Antonino Casabona, Sara Lanza, Rosario Marchese-Ragona, Francesco Patti, Stefano Masiero, Antonio Biondi, Angelo Quartarone

**Affiliations:** 1 Neurology Department, Garibaldi Hospital, 95100 Catania, Italy; 2 Department of Molecular and Clinical Pharmacology, Institute of Translational Medicine, University of Liverpool, Liverpool L271XF, UK; dott_mariangela@hotmail.com; 3 Department of Biomedical and Biotechnological Sciences, Section of Physiology, University of Catania, 95100 Catania, Italy; casabona@unict.it; 4 UOC di Medicina Fisica e Riabilitazione, Comiso-Vittoria, ASP Ragusa, 97013 Ragusa, Italy; sara.lanza@asp.rg.it; 5 ENT Department, University of Padova, 35121 Padova, Italy; rossmr@libero.it; 6 DANA Department, “GF Ingrassia”, Neuroscience Section—Multiple Sclerosis Center, University of Catania, 95100 Catania, Italy; patti@unict.it; 7 School of Physical Medicine and Rehabilitation, University of Padua, 35121 Padua, Italy; stef.masiero@unipd.it; 8 Department of Surgery, University of Catania, 95100 Catania, Italy; abiondi@unict.it; 9 IRCCS Centro Neurolesi “Bonibo-Pulejo”, via Provinciale Palermo, Contrada Casazza, 95124 Messina, Italy; aquartar@unime.it or aquartar65@gmail.com

**Keywords:** sialorrhoea, drooling, salivary glands, swallowing, botulinum toxin, eccrine glands, *onabotulinumtoxin A*, *incobotulinumtoxin A*

## Abstract

*Background*: Sialorrhoea and drooling are disabling manifestations of different neurological disorders. The aim of this study was to evaluate the effects of botulinum neurotoxin type A (BoNT/A) injection on hypersalivation in 90 patients with neurological diseases of different aetiologies, and to define the minimum number of injected salivary glands to reduce sialorrhoea. Determining the minimum number of glands that need to be engaged in order to have a significant reduction in drooling may be very useful for establishing the minimum total dosage of BoNT/A that may be considered effective in the treatment of hypersalivation. *Methods*: Twenty-five mouse units (MU) of BoNT/A (*onabotulinumtoxin A,* Botox; Allergan, Irvine, CA, USA; 100 MU/2 mL, 0.9% saline; or *incobotulinumtoxin A*, Xeomin; Merz Pharma, Germany; 100 MU/2 mL, 0.9% saline) were percutaneously injected into the parotid (p) glands and/or submandibular (s) glands under ultrasound control. On this basis, patients were divided into three groups. In group A (30 patients), BoNT/A injections were performed into four glands; in group B (30 patients), into three glands, and in group C (30 patients), into two glands. Patients treated in three glands (group B) were divided into two subgroups based on the treated glands (2 p + 1 s = 15 patients; 2 s + 1 p = 15 patients). Similarly, patients being injected in two glands (group C) were subdivided into three groups (2 p = 10 patients; 1 p + 1 s = 10 patients; 2 s = 10 patients). In patients who were injected in three and two salivary glands, saline solution was injected into the remaining one and two glands, respectively. Assessments were performed at baseline and at 2 weeks after the injections. *Results*: BoNT/A significantly reduced sialorrhoea in 82 out of 90 patients. The effect was more evident in patients who had four glands injected than when three or two glands were injected. The injections into three glands were more effective than injections into two glands. *Conclusions*: Our results have shown that BoNT/A injections induced a significant reduction in sialorrhoea in most patients (91%). In addition, we demonstrated that sialorrhoea associated with different neurological diseases was better controlled when the number of treated glands was higher.

## 1. Introduction

Sialorrhoea or hypersalivation and drooling are known to be associated with several neurological disorders [1]. Other than a disabling social problem, hypersalivation is often associated with impairment of swallowing coordination. This condition affects about 10% of patients with cerebral palsy and post-traumatic encephalopathy [2] and 20% of patients with amyotrophic lateral sclerosis [3]. In Parkinson’s disease, its frequency varies from 10% to 84% [4].

Salivation is controlled by the autonomic nervous system via cholinergic nerve fibres. In healthy adults, the parotid (p) and submandibular (s) glands—the major salivary glands—account for about 95% of the total salivary secretion. The remaining 5% is produced by the lingual and minor glands [5].

Traditional treatment of excessive drooling includes anticholinergic oral drugs, surgical intervention and local irradiation of salivary glands [6,7]. However, these treatments are poorly tolerated, are invasive and are often ineffective in a number of patients. In fact, systemic anticholinergic drugs are often ineffective and produce unacceptable side effects such as blurred vision, urinary retention, and cardiac arrhythmia. Surgical intervention and local irradiation of salivary glands have been performed, but these are invasive procedures that are often unacceptable to patients and their caregivers.

Recently, the percutaneous injection of botulinum neurotoxin type A (BoNT/A) into salivary glands has been shown to be effective in abolishing excessive sialorrhoea associated with several neurological disorders [8,9,10,11,12,13]. In addition, studies on botulinum neurotoxin type B (BoNT/B) in patients affected with cervical dystonia have shown a relatively high incidence of dry mouth [14]. This suggests that BoNT/B may be more effective in the treatment of hypersalivation than BoNT/A [14]. However, in our country, BoNT/B treatment can be performed only in patients unresponsive to BoNT/A.

The rationale for the use of BoNT/A in this condition is the selective block of presynaptic release of acetylcholine from the cholinergic endings supplying eccrine salivary glands [15,16]. Usually, the effect of this treatment lasts several months. Although encouraging, the results of these studies were obtained from a restricted number of subjects. Moreover, in previous studies, a correlation between the number of glands treated and the amount of salivation reduction was not investigated.

The aim of the present study was thus to evaluate the effects of BoNT/A injection on hypersalivation in a wide number of patients with different neurological disorders and, in addition, to evaluate whether the number of treated glands might influence the efficacy of the treatment. The determination of the minimum number of glands that should be engaged in order to have a significant drooling reduction may be very useful for establishing the minimum total dosage of BoNT/A that may be considered effective in the treatment of hypersalivation.

## 2. Results

In evaluating the effects of BoNT/A, we detected four different levels of responsiveness to the treatment. We scored from 3 (good responders with a reduction of 75% in the roll weight) to 0 (no response; when weights of the wet rolls did not change); intermediate and poor responders were scored with 2 or 1, respectively, when the reduction of their roll weights was equal to 50% or 25% (see Table 1).

**Figure 1:**
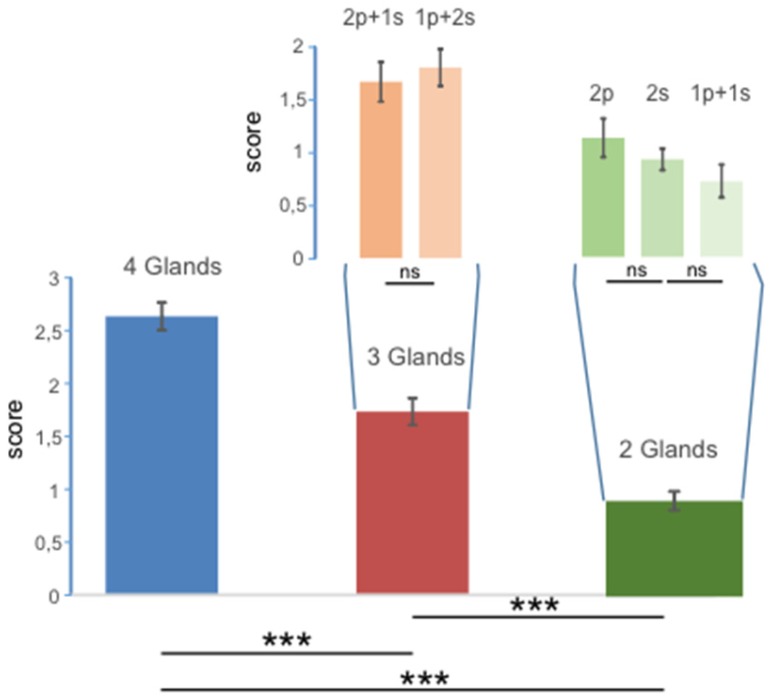
Values of scores obtained over the groups with injections into four, three and two glands (large bars) and in the groups where injections were unevenly distributed between parotid (p) and submandibular (s) glands (narrow bars). The error bars represent the standard error, *** indicates differences with *p* < 0.00001, and ns indicates no statistical differences.

Eighty-two out of 90 patients (91%) were responders to the treatment. In almost all patients, sialorrhoea was dramatically reduced at 2 weeks (mean dental roll weight before BoNT/A: 0.8 ± 0.08 g; mean dental roll weight 2 weeks after BoNT/A: 0.25 ± 0.1 g). Only 8 (8.8%) out of 90 patients did not benefit from the treatment. Patients with injections into four, three and two glands showed large differences over the three groups (Figure 1 F_2,87_ = 48.78; *p* < 0.000001), with the highest average score being observed for the four-gland group (2.63 ± 0.13) and lower scores recorded for the three-gland group (1.73 ± 0.13) and for the two-gland group (0.9 ± 0.09). Significant differences were also observed for each paired comparison (four glands vs. three glands: *p* = 0.000003; four glands vs. two glands: *p* < 0.000001; three glands vs. two glands: *p* < 0.000007).

The narrow bars inserted in Figure 1 represent the scores recorded when injections were unevenly distributed between parotid and submandibular glands. No significant differences were detected and all the scores were similar regardless of which glands were most engaged.

**Figure 2:**
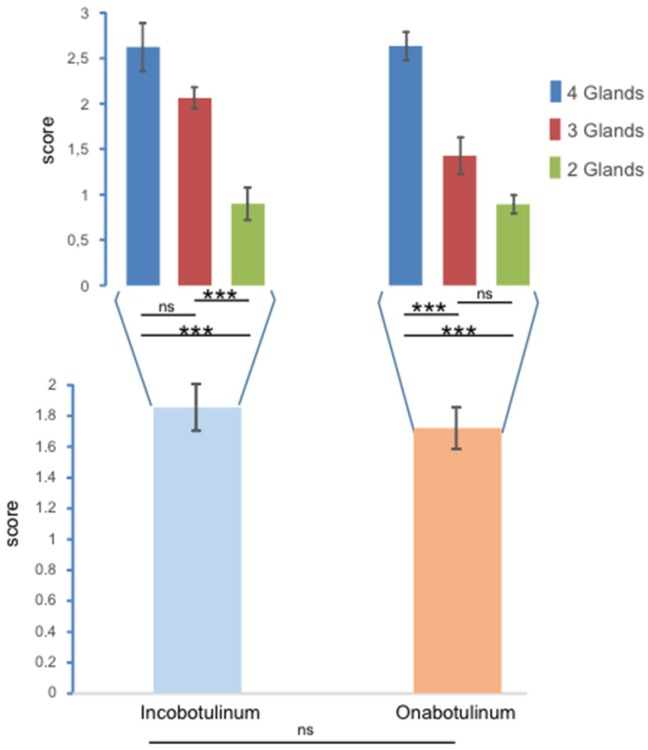
Values of scores observed with respect to the two types of toxins injected (incobotulinum and onabotulinum). Large bars represent the results obtained from the entire sample of patients; the narrow bars show the subdivision over the groups with injections into four, three and two glands. The error bars represent the standard error, *** indicates differences with *p* < 0.00001, and ns indicates no statistical differences.

Although the score for *incobotulinum toxin* was higher than the score for *onabotulinumtoxin*, the type of toxin did not produce significant differences when the score was measured over the entire sample of patients (N = 90; F_1,84_ = 2.47; *p* = 0.12), and no interaction was observed between the number of glands engaged and the type of toxin (F_2,84_ = 2.76; *p* = 0.069). This is reported in Figure 2.

When the dataset was separated into two subsamples associated with each toxin, significant differences were observed over the three groups for both the effect of *incobotulinumtoxin* (F_2,30_ = 22.45; *p* < 0.000001) and *onabotulinumtoxin* (F_2,54_ = 40.14; *p* < 0.000001). The largest influence was detected in group A and, except for the comparison between group A and group B for *incobotulinumtoxin* and between group B and C for *onabotulinumtoxin*, the other paired comparisons were statistically different.

The overall effect of disease was statistically significant (F_4,85_ = 6.68; *p* < 0.0001). The Bonferroni multiple comparison showed that the main contribution to this result was provided by the paired differences between ALS and CP (*p* = 0.0033), PD (*p* < 0.0001) and stroke (*p* = 0.0022).

Only one patient complained of dysphagia 7 days after BoNT/A injection, which disappeared within 15 days; another two patients had a hematoma at the site of injection, which needed compressive bondage for 2 h.

## 3. Discussion

This is the first randomized, blinded study exploring the relationship between the effect of BoNT/A injections and the number of salivary glands injected.

Our results showed that BoNT/A injections induced a dramatic reduction of sialorrhoea in almost all patients (91%). The reduction of sialorrhoea was evident 2 weeks after the injection. However, the production of saliva was still enough that swallowing of both foods and drink in all patients was not impaired. In fact, parotid and submandibular glands together account for about 95% of the total salivary secretion. The remaining 5% is produced by the lingual and minor glands [5].

The effect of salivation reduction was more significant in patients with four glands treated than patients with three or two glands treated. Patients treated in three glands showed a more significant reduction in hypersalivation than patients treated in two glands. In patients treated in three glands, no significant differences were observed between injections into one parotid and two submandibular glands and injections into one submandibular gland and two parotid glands. In patients treated in two glands, no significant differences were observed among patients who were injected into two parotids, two submandibular glands or one parotid and one submandibular gland, respectively.

Otherwise, we cannot exclude that the beneficial effect was due to the different total doses that were used. Both dose and number of injected glands may have a synergic effect in reducing hypersalivation.

It is noteworthy that the score recorded for the *incobotulinumtoxin A* tended to be higher than the *onabotulinumtoxinA,* and the two toxins showed slightly different effects over the groups. In fact, as both of the toxins influenced the score of patients treated in four and two glands, *incobotulinumtoxin A* differentiated the scores associated with patients treated in three and two glands, while *onabotulinumtoxin A* exhibited an effect between patients treated in four and three glands. Thus, the overall influence of BoNT/A derived from a differentiated effect of the single toxins.

The major number of injected glands did not produce any side effects and the dose of BoNT/A used for each gland was safe in all patients. This treatment has the advantage that it avoids the use of oral anticholinergic medications, which has previously been administered in most patients for the symptomatic therapy of the sialorrhoea.

However, this study did not establish whether BoNT/A parotid injections are superior to BoNT/A submandibular injections in reducing hypersalivation. In fact, when only two parotids or two submandibular glands were injected (Group C, 2p and 2s patients, respectively), no significant differences were observed.

In addition, our findings have shown that injections in patients with sialorrhoea associated with ALS were less effective than in patients with PD, stroke, BI or CP. A possible explanation for these differences may reside in a different saliva composition (more prevalence of mucinoses component in the saliva of patients with ALS) or with a more impaired oral/preparatory phase of swallowing in ALS, with consequent major saliva pooling in the mouth. However, further studies specifically focused on this topic and involving a larger number of ALS patients are needed before drawing definitive conclusions.

Our study demonstrated that sialorrhoea due to different neurological diseases may be successfully managed with injections into four or at least three salivary glands. This treatment is safe, simple and highly effective.

## 4. Materials and Methods

### 4.1. Patients

**Figure 3:**
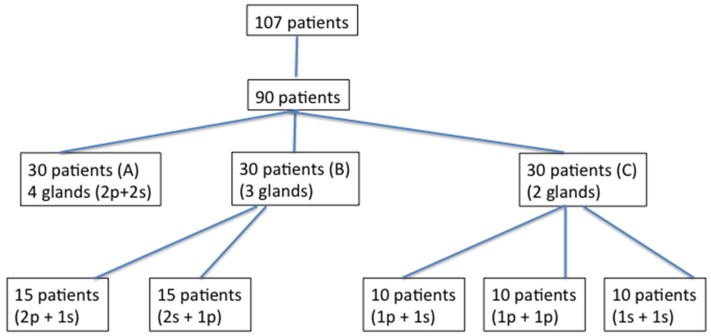
Study profile.

A consecutive series of 117 patients with neurological disorders arising from different aetiologies was screened in our hospital from January 2005 to December 2016 for hypersalivation. Out of these 117 patients with sialorrhoea, ninety subjects (59 men and 31 women; mean age: 53.4 ± 17.6 years) who had experienced a high frequency and severity of hypersalivation and drooling in the preceding 6 months satisfied all of the inclusion/exclusion criteria and were enrolled in the study (Figure 3). We included those patients who had wet rolls with a roll-weight at least ten times heavier than the dry rolls. Thirty out of 90 patients were affected by Parkinson disease (PD), 11 were suffering from amyotrophic lateral sclerosis (ALS), 21 were affected by stroke, 8 by brain injury (BI) and 20 by cerebral palsy (CP). Table 1 shows the demographic characteristics of these 90 patients.

### 4.2. Inclusion criteria

(1) Accepting to participate in the study; (2) age ≥ 18 and ≤ 75 years; (3) the diagnosis of one of the following neurological disorders often associated with hypersalivation and/or drooling: diagnosis of PD, stroke, ALS, CP, or BI; (4) severely disabling sialorrhoea lasting for at least 6 months.

### 4.3. Exclusion criteria

The presence of other neurological diseases or the inability to give informed consent because of cognitive impairment.

All patients gave their written informed consent to the BoNT/A treatment after the approval of the local ethics committee.

### 4.4. Treatment

Twenty-five mouse units (MU) of botulinum neurotoxin type A (BoNT/A; Botox, [*onabotulinumtoxin A*], Allergan, Irvine, CA, USA; 100 MU/2 mL, 0.9% saline; or twenty-five mouse units (MU) of BoNT/A, Xeomin [*incobotulinumtoxin A*], Merz Pharma, Frankfurt, Germany; 100 MU/2 mL, 0.9% saline) were injected into each parotid gland using a 27G 3/4 needle. The submandibular glands were also injected with 25 MU each. Out of 90 patients, 57 patients were treated with *onabotulinumtoxin A* and 33 patients with *incobotulinumtoxin A*. The total number of patients treated with *onabotulinumtoxin A* injections was more than the number of patients treated with *incobotulinumtoxin A* because the latter was introduced later on. BoNT/A was percutaneously injected in all patients into the salivary glands under ultrasound control using a linear electronic probe 7.5 MHz (Aloka 650-SSD), which allowed the operator to accurately inject into the targeted salivary gland. Injections were performed once in all patients. Before injection, patients underwent an objective assessment of sialorrhoea and then they were randomised into three groups according to the number of treated glands. The number of glands that were injected was randomly assigned using a computer-generated list. Patients were divided into three groups (group A, B, and C). In group A (N = 30 patients; 10 females and 20 males; age range: 18–73 years), BoNT/A injections were performed into four glands (2p + 2s); in group B (N = 30 patients; 12 females and 18 males; age range: 18–72 years), BoNT/A injections were performed into three glands (2p + 1s, N = 15 patients; 2s + 1p, N = 15 patients) and in group C (30 patients; 10 females and 20 males; age range: 21–73 years), BoNT/A was injected into two glands (1p + 1s, N = 10 patients; 2p, N = 10 patients; 2s, N = 10 patients). In patients with four salivary glands treated, the total dose of botulinum toxin injected was 100 MU (25 UI for each gland). In patients with three and two salivary glands injected, the total dose was 75 MU and 50 MU (25 MU per gland), respectively. In patients injected into three and two salivary glands, an equal amount of saline solution was injected into the remaining one and two glands, respectively.

### 4.5. Assessment

Assessments were made at the same time of the day for each visit and patients were seated upright. Patients were inhibited from eating or drinking one hour before each assessment. Assessments were performed by a physician examiner who was blinded to the treatment allocation at baseline, and then at 2 weeks after the injection. All patients were blinded to their treatment allocation. Both treating and physician examiners reported their evaluations in separate case report forms. Hypersalivation was measured with six dental rolls placed in six different areas of the mouth and then retained there for 5 min. The difference in weight between the dry and wet rolls was calculated. The procedure was repeated after 15 min. The mean value of these consecutive assessments was taken as the final value.

### 4.6. Statistical analysis

Likert’s transformation was obtained to change the qualitative results of BoNT/A injection into salivary glands into a score ranging from 0 (no effect) to 3 (maximum effect = 75% reduction of sialorrhoea). Two-way analysis of variance (ANOVA) was performed to evaluate the effect of the number of glands receiving toxin injection and the type of toxin. One-way ANOVA was applied to the two subgroups with injections into three glands (2p + 1s, 1p + 2s; N = 15 for each group) and the three subgroups with injections into two glands (2p, 2s, 1p + 1s; N = 10 for each group). An additional one-way ANOVA was used to estimate the effect of disease on the score value. For the ANOVA with three levels, the multiple comparisons were adjusted by Bonferroni correction. The data are expressed as means and standard errors, and the results were considered significant when *p* < 0.05. Statistical analyses were performed using SYSTAT version 11 (Systat Inc., Evaston, IL, USA).

## Figures and Tables

**Table 1:** The demographic characteristics of the 90 neurological patients with hypersalivation treated with BoNT/A injections into different salivary glands. Abbreviations: PD, Parkinson disease; ALS, amyotrophic lateral sclerosis; BI, brain injury; CP, cerebral palsy; s, submandibular; p, parotids; Inco = incobotulinum toxin A; Ona = onabotulinumtoxin A.

Patient	Age	Sex	Disease	Injected Glands	Effects (2 Weeks)	Type of Toxin	Group
1	40	F	CP	2p-2s	2	Ona	A
2	56	F	PD	2p-2s	3	Inco	A
3	73	M	PD	2p-2s	3	Inco	A
4	71	M	PD	2p-2s	3	Inco	A
5	67	F	Stroke	2p-2s	2	Ona	A
6	72	M	Stroke	2p-2s	3	Ona	A
7	63	M	PD	2p-2s	3	Ona	A
8	65	F	PD	2p-2s	3	Ona	A
9	50	M	BI	2p-2s	2	Ona	A
10	28	M	Stroke	2p-2s	3	Ona	A
11	55	M	PD	2p-2s	3	Ona	A
12	55	F	Stroke	2p-2s	3	Ona	A
13	67	F	PD	2p-2s	3	Ona	A
14	72	M	PD	2p-2s	2	Ona	A
15	63	M	PD	2p-2s	3	Ona	A
16	65	F	Stroke	2p-2s	2	Ona	A
17	73	M	PD	2p-2s	3	Ona	A
18	71	M	PD	2p-2s	3	Inco	A
19	67	M	BI	2p-2s	1	Inco	A
20	55	M	PD	2p-2s	3	Inco	A
21	63	M	PD	2p-2s	3	Ona	A
22	65	M	PD	2p-2s	3	Ona	A
23	50	F	Stroke	2p-2s	3	Ona	A
24	45	F	CP	2p-2s	3	Ona	A
25	19	F	CP	2p-2s	3	Ona	A
26	71	M	PD	2p-2s	0	Ona	A
27	72	M	PD	2p-2s	2	Inco	A
28	66	M	Stroke	2p-2s	3	Inco	A
29	18	M	BI	2p-2s	3	Ona	A
30	18	M	CP	2p-2s	3	Ona	A
31	61	M	PD	2p-1s	1	Ona	B
32	59	M	ALS	2p-1s	0	Ona	B
33	71	M	PD	2p-1s	2	Ona	B
34	48	M	PD	2p-1s	1	Ona	B
35	55	M	BI	2p-1s	2	Inco	B
36	67	M	PD	2p-1s	2	Inco	B
37	72	F	Stroke	2p-1s	2	Inco	B
38	55	F	ALS	2p-1s	2	Inco	B
39	63	M	Stroke	2p-1s	2	Ona	B
40	65	M	Stroke	2p-1s	2	Ona	B
41	50	M	PD	2p-1s	1	Ona	B
42	67	F	PD	2p-1s	2	Inco	B
43	70	M	PD	2p-1s	2	Inco	B
44	53	M	PD	2p-1s	3	Inco	B
45	22	M	CP	2p-1s	1	Ona	B
46	53	M	ALS	1p-2s	1	Inco	B
47	47	M	Stroke	1p-2s	2	Inco	B
48	59	F	PD	1p-2s	2	Inco	B
49	72	M	Stroke	1p-2s	2	Inco	B
50	61	F	PD	1p-2s	1	Ona	B
51	18	F	CP	1p-2s	2	Inco	B
52	21	M	CP	1p-2s	2	Inco	B
53	18	F	CP	1p-2s	2	Ona	B
54	69	M	PD	1p-2s	2	Inco	B
55	49	F	CP	1p-2s	3	Inco	B
56	29	F	CP	1p-2s	2	Ona	B
57	46	F	CP	1p-2s	2	Ona	B
58	68	F	ALS	1p-2s	0	Ona	B
59	29	F	CP	1p-2s	2	Ona	B
60	57	M	Stroke	1p-2s	2	Ona	B
61	66	F	ALS	2p	0	Inco	C
62	38	M	BI	2p	1	Ona	C
63	66	F	ALS	2p	1	Ona	C
64	38	M	BI	2p	1	Ona	C
65	36	M	CP	2p	2	Ona	C
66	28	M	CP	2p	1	Inco	C
67	63	M	ALS	2p	1	Ona	C
68	71	F	Stroke	2p	2	Inco	C
69	70	M	ALS	2p	1	Ona	C
70	69	M	Stroke	2p	1	Ona	C
71	58	M	Stroke	2s	1	Inco	C
72	61	M	PD	2s	1	Inco	C
73	70	F	ALS	2s	1	Inco	C
74	65	F	ALS	2s	0	Ona	C
75	25	F	Stroke	2s	1	Ona	C
76	22	M	CP	2s	1	Ona	C
77	37	M	BI	2s	1	Inco	C
78	54	M	BI	2s	1	Ona	C
79	70	M	Stroke	2s	1	Ona	C
80	59	M	Stroke	2s	1	Ona	C
81	22	M	CP	1p-1s	1	Ona	C
82	24	M	CP	1p-1s	1	Inco	C
83	49	F	ALS	1p-1s	0	Inco	C
84	21	M	CP	1p-1s	1	Ona	C
85	73	M	PD	1p-1s	1	Inco	C
86	69	F	PD	1p-1s	0	Ona	C
87	26	F	CP	1p-1s	1	Ona	C
88	68	M	Stroke	1p-1s	1	Ona	C
89	65	F	Stroke	1p-1s	0	Ona	C
90	34	M	BI	1p-1s	1	Ona	C
